# Progress in ileal endogenous amino acid flow research in poultry

**DOI:** 10.1186/s40104-020-00526-2

**Published:** 2021-01-06

**Authors:** V. Ravindran

**Affiliations:** Monogastric Research Centre, School of Agriculture and Environment, Massey University, Palmerston North, 4442 New Zealand

**Keywords:** Basal loss, Digestible amino acids, Endogenous amino acid losses, Poultry, Specific loss

## Abstract

The progress in our understanding of the endogenous protein concept over the past century is reviewed. Non-dietary proteins found in the digesta at the terminal ileum of poultry, known as endogenous protein loss, are comprised of digestive secretions, mucus and sloughed gut epithelial cells. The measurement of this loss is of fundamental importance because it is an indicator of gut metabolism and is essential to adjust apparent estimates of ileal amino acid digestibility. The ileal endogenous amino acid losses comprise of two components, namely basal and specific losses. The basal losses are fixed and associated with feed dry matter intake, whereas the specific losses are variable and induced by the presence of dietary components such as fibre and anti-nutrients. Currently there is no methodology available to directly measure the specific endogenous losses and these losses are calculated by determining the basal and total (basal plus specific) losses and, then subtracting the basal losses from total losses. The seminal features, specific applications and shortcomings of available methodologies are briefly outlined as well as the practical challenges faced in using the published endogenous amino acid loss values for true digestibility corrections. The relevance of taurine as a component of endogenous protein flow in poultry is identified for the first time.

## Introduction

Endogenous protein loss, which links the gut physiology and protein nutrition, has proved to be an alluring subject of research for many years. Historically, the presence of undigested substances of digestive tract origin in the excretory products of animals and its relevance to the biological value of protein has long been known [1–3]. In poultry, the earliest work on the use of nitrogen-free diets was by Ackerson et al. [4] to study the variation in nitrogen excretion patterns with time and moulting. Bragg et al. [5] are credited with the first report of the quantification of endogenous amino acid losses in poultry and using them as correction factors in digestibility calculations. In their study, a nitrogen-free diet was fed to measure the metabolic and endogenous losses in colostomised birds over the total digestive tract.

Over the past two decades, the measurement of amino acid digestibility of ingredients for poultry has shifted to ileal level from the excreta, because of the variable and modifying effects of the hindgut microbiome on protein utilisation and the contribution of metabolic nitrogen due to urine contamination [6]. Currently the use of ileal digestibility values in feed formulation is the norm in the poultry industry and therefore the current review will focus on ileal endogenous losses instead of early research on metabolic and endogenous flow measurements at the excreta level.

During the digestion of ingesta, there is a continuous input of endogenous proteins, in the form of secretion of digestive enzymes and bile, shedding of mucus and desquamated enterocytes, into the lumen of the intestine. This input of endogenous proteins is copious and estimated to be as much as four- or five-fold of the amount of ingested proteins [7]. These endogenous sources mix with dietary protein and are digested, and the resulting amino acids are absorbed. The magnitude of reabsorption of endogenous proteins up to the ileal level in poultry is not known. In pigs, Souffrant et al. [8] estimated that 79% of the gross endogenous secretion in pigs is reabsorbed, but the degree of re-absorption varies depending on the relative ratio of individual endogenous components and their point of entry into the gut, being high with digestive enzymes and lower with mucin. Endogenous protein flow measured at the terminal ileum, therefore, represent the net balance between ingested protein and endogenous protein secretions minus absorption of dietary protein and reabsorption of endogenous protein [9]. The unabsorbed endogenous portion that passes beyond the ileum is fundamentally lost to the animal and referred to as inevitable, obligatory losses.

Accurate quantification of endogenous amino acid (EAA) losses is important for several reasons. First, the correction for these unavoidable losses is required for the calculation of true amino acid digestibility of feed ingredients. Second, it is necessary for the determination of protein and amino acid requirements by the factorial method. Third, these losses have significant ramifications in terms of the metabolic cost associated with protein synthesis and turnover in the gut. Finally, the measurement of these losses and better understanding of the factors by which they are influenced provide an effective strategy to improve the efficiency of protein and amino acid utilisation.

It is recognised that EAA losses are influenced primarily by feed intake and secondarily by the inherent composition of the feed ingredient or diet. These two fractions are conveniently categorised to as basal (also known as non-specific) and specific EAA losses, respectively [10]. Basal endogenous losses are defined as those inevitable losses closely associated with the metabolic functions of the animal and are independent of the diet type. These losses represent the minimum losses that can be expected under any feeding situation. Specific losses, in contrast, are diet-related resulting from the presence of specific dietary components such as sources and amounts of fibre and various anti-nutritional factors (e.g. phytate, trypsin inhibitor, lectins etc.). Currently there is no methodology available to directly quantify the specific EAA losses. These losses, however, can be calculated by determining the total (basal plus specific) losses and then subtracting the basal losses from total losses.

The microcosm of endogenous protein flow is too extensive to comprehensively review herein and it is necessary, therefore, to treat the subject selectively. The review of Sibbald [11] provides an excellent coverage of early research on metabolic and endogenous losses at the excreta level in poultry. Subsequent reviews have focused on ileal endogenous losses and several exhaustive contributions are available [12–16], which provide background information for the present paper. In the current review, it seems appropriate to focus on the progress made on the measurement of ileal EAA in poultry and on the understanding of factors influencing EAA losses, followed by challenges faced by the industry in translating the endogenous flow data into practical application.

## Sources of endogenous secretions

The first step in a discussion on EAA flow should be what constitutes endogenous proteins. These proteins originate predominantly from various digestive secretions (bile, pancreatic enzymes and, gastric and intestinal secretions), mucoproteins and desquamated intestinal epithelial cells. Microbial mass also contributes significantly to the nitrogen found in the ileal digesta [17, 18], but paradoxically not considered neither as endogenous nor as dietary origin.

As mentioned, EAA flow determined at the terminal ileum is the net result of the digestive dynamics of endogenous sources along the digestive tract and represents the algebraic difference between that secreted and absorbed. The source of endogenous proteins and the entry point into the digestive tract appears to be critical in determining this dynamic. When digestive enzymes dominate the endogenous flow, the proteins pass through the duodenum and jejunum where there is greater opportunity for digestion and absorption. In contrast, if mucus secretion or desquamation is significant, particularly if they occur distal to the duodenum, then the opportunity for digestion is lower and relatively higher endogenous losses will be inevitable at the ileal level. Owing to this complicated nature of digestion trends, it is difficult to assess the true contribution of each endogenous protein source.

Interestingly, studies with poultry have shown that the amino acid composition of ileal endogenous protein is generally constant, independent of diet and method of determination. As shown in Table 1, the class of chicken (broilers, layers or roosters) has little influence on the EAA profile and, the profiles of chickens and pigs are remarkably similar.

**Table 1:** Amino acid composition of basal ileal endogenous protein (g/100 g crude protein) in different classes of poultry, in comparison to those reported in pigs (all measured using the enzyme hydrolysed casein method)

Amino acid^a^	Broilers^b^	Layers^b^	Roosters^b^	Growing pigs^c^
Aspartic acid	7.2	7.4	7.9	7.7
Threonine	6.3	7.5	7.0	5.1
Serine	6.0	6.2	8.3	6.7
Glutamic acid	13.7	10.5	15.1	16.5
Proline	7.8	6.4	6.6	11.8
Glycine	5.2	5.8	6.2	7.8
Alanine	3.4	5.2	3.1	3.7
Valine	4.6	4.9	5.5	4.0
Isoleucine	3.4	3.3	4.7	3.4
Leucine	4.7	4.9	4.7	4.1
Tyrosine	2.6	2.7	2.3	2.2
Phenylalanine	3.4	3.4	2.9	2.4
Histidine	2.3	2.4	2.2	2.0
Lysine	3.0	3.2	3.6	3.0
Arginine	3.0	3.0	2.5	2.7
Methionine	1.3	1.2	1.1	–
Cysteine	2.0	2.3	2.1	–

^a^The order of listed amino acids reflects the order of elusion in the Shimatzu LC-MS and Dionex HPLC system used in our labs. It must be noted that the order of elution will change depending on depending on methods and conditions

^b^Ravindran and Hendriks [19]

^c^Jansman et al. [20]

The most abundant amino acids in the ileal endogenous protein of both species were glutamic acid, aspartic acid, threonine, proline, serine and glycine. These amino acids are found in high concentrations in intestinal and pancreatic secretions, and mucoproteins (Table 2), confirming that these are the major components of endogenous protein.

**Table 2:** Amino acid profile (g/100 g amino acids) of different endogenous secretions

Amino acid	Pancreatic secretions^a^	Bile (pigs)^b^	Bile (broilers)^c^	Mucin^d^
Aspartic acid	12.5	0.4	2.1	7.8
Threonine	5.2	0.3	1.5	16.4
Serine	6.9	0.3	1.6	10.9
Glutamic acid	10.3	1.1	3.2	10.1
Proline	5.0	0.3	1.4	12.0
Glycine	6.2	95.0	1.9	5.5
Alanine	5.4	0.0	1.1	7.4
Valine	7.2	0.3	1.7	5.9
Isoleucine	5.9	0.2	1.0	3.0
Leucine	8.3	0.4	1.8	5.7
Tyrosine	5.7	0.2	1.1	3.2
Phenylalanine	4.4	0.2	1.0	3.5
Histidine	2.6	0.2	0.8	1.7
Lysine	5.1	0.3	1.2	2.8
Arginine	5.1	0.3	2.5	3.5
Taurine	–	–	73.7	–
Methionine	1.1	0.1	0.7	0.8
Cysteine	1.7	0.6	1.7	10.0

^a^Pancreatic secretions, pigs; Corring and Jung [21]

^b^Juste [22]

^c^Massey University, unpublished data

^d^Mucin from the small intestine, pigs; Lien et al. [23]

Threonine, proline, serine and glutamic acid predominate intestinal mucus glycoproteins [24], indicating greater presence of mucins, which are thought to be resistant to enzymatic hydrolysis, in the ileal protein flow [25]. Glycine, the abundant amino acid in biliary secretions of pigs, escapes reabsorption as deconjugated glycine [26] and contributes to the high glycine content of endogenous protein. However, this is not the case in poultry where the bile acid conjugation is almost exclusively with taurine [27]. In all poultry studies, the high concentration of glycine in EAA is attributed to bile secretions [13, 14, 19]. None of the EAA data in poultry to date have considered or reported taurine values. Clearly in future EAA studies with poultry, taurine estimates must be included, and this will give important information on the contribution of bile secretions to ileal endogenous losses.

Depending on the amount and type of fibre and specific anti-nutrient, the effect on components of endogenous secretion differ. For example, the effect of trypsin inhibitor appears to be largely on increasing pancreatic secretions [28], whereas that of insoluble fibre may be mainly on mucin and of soluble, viscous fibre may possibly have a major effect of bacterial component [12].

## Measurement of basal endogenous protein losses

The measurement and relevance of basal EAA losses in protein nutrition are dealt elsewhere [14, 15]. The basal EAA estimates are needed to calculate the specific losses from total losses.

A summary of various available methodologies for the quantification of basal losses is presented in Table 3. Of these, feeding of a protein-free diet is the most widely used method because of its simplicity. The basic assumption is that a meal devoid of protein still provides adequate stimulus in the gastrointestinal tract to secrete endogenous proteins. It must be recognised, however, that the absence of protein will profoundly change nitrogen balance and whole-body protein synthesis, and the animal no longer could be considered as physiologically normal.

**Table 3:** Methods used for the determination of basal and total endogenous amino acid losses in poultry

Item	Methods
Basal losses	• Fasting of birds for 48 h^a^
	• Feeding of a protein-free diet
	• Feeding of diets with very low levels of protein
	• Protein-free diet supplemented with synthetic amino acids
	• Protein-free diets with intravenous amino acid infusion
	• Natural proteins devoid of specific amino acids
	• Enzyme hydrolysed casein^b^ and ultrafiltration
	• Feeding of highly digestible protein, e.g. wheat gluten, soy protein isolate, casein^b^
	• Linear regression, following feeding of diets containing graded levels of specific ingredient
Total losses	• Homoarginine
	• Isotope-labelled markers

^a^Specific to precision-fed rooster assays [29, 30] and measured in the excreta. Measurement units are function of time (mg/bird/d) rather than intake (mg/kg dry matter intake) and cannot be used as correction factor in other digestibility assays

^b^These proteins are assumed to be (almost) 100% digestible and that excreted amino acids therefore represent endogenous losses

In recent decades, with the increasing acceptance of ileal-based digestibility concept, the site of measurement of endogenous flow has been moved from the excreta to ileal level [31]. In broiler chickens, basal ileal EAA losses have been reported to range from 3.08 g/kg dry matter intake (DMI) for the protein-free diet to 8.81 g/kg DMI for the highly digestible protein method [32]. The reliability of available methods, under a given set of dietary conditions, had been an impediment in the measurement of basal EAA losses and, as with most biological responses, the perfect technique for measuring EAA losses is yet to be developed.

## Measurement of total endogenous protein losses

Two methodologies, namely isotopic and guanidination techniques, are available for the measurement of total endogenous losses.

### Techniques involving isotope markers

Substantial research investment was made during the 1980’s and 1990’s to measure the total EAA in pigs using either stable (^15^N) or radioactive (^14^C, ^35^S, ^75^Se) isotopes. The ^15^N isotope dilution technique of Souffrant et al. [33] was particularly popular and employed by a number of researchers [34, 35] to differentiate endogenous proteins from non-digested dietary proteins in the ileal digesta of pigs. The method involves the perfusion of ^15^N-labelled leucine into the blood of pigs consuming the test protein. The labelled amino acid is incorporated in the endogenous secretions and the dilution of ^15^N measured in the digesta gives an estimation of the amount of endogenous nitrogen. The data from isotope dilution demonstrated that the recovery of endogenous proteins in the ileal digesta was higher than those determined by feeding a protein-free diet. Most of these isotopic studies have been conducted with pigs and only two with poultry [36, 37].

Though attractive, this technique suffers from several constraints, because the determination of ^15^N enrichment of endogenous secretions is demanding and burdensome. The inability to measure the recovery of all individual amino acids in ileal digesta [34, 38] and the rapid precursor pool recycling [33] are other drawbacks. These limitations have been elegantly discussed by Leterme et al. [39] and Moughan et al. [40]. In particular, standardisation of conditions such as feeding frequency, diet type, infusion protocol, rate of tracer infusion, sampling procedures, sample preparation and choice of precursor pool(s) is imperative if reliable comparisons of data from different laboratories are to be made [41].

Xu et al. [42] described an alternative isotopic method involving a single muscular injection of ^3^H-labelled leucine to measure EAA losses in broilers. It was stated that this novel approach overcomes most of the limitations of isotope dilution techniques and, according to Makkar [43], is thought to be as effective.

### Homoarginine technique

An innovative approach, using homoarginine (HA) as a marker, to determine EAA losses was proposed by Hagemeister and Erbersdobler [44]. In this method, ε-amino group of lysine in dietary proteins are converted into HA by the guanidination reaction, involving treatment with O-methylisourea under alkaline conditions. After the labelled protein is fed, endogenous losses of amino acids are determined by comparing amino acid: HA ratios in the diet and ileal digesta. Homoarginine is not found in normal feedstuffs. It is digested and absorbed in a manner like other amino acids [31], but does not reappear in endogenous secretions into the gut and this unique feature enables the calculation of total EAA losses.

The HA method is based on following premises, most of which have been tested and found to be tenable, and have been explored at length [13, 40, 45–47].

1. Absorbed HA is not incorporated into tissue proteins and therefore not re-cycled into the intestine [48].
2. The guanidinated protein is homogeneously and randomly labelled with HA, a premise proven to be true for purified proteins [31].
3. Homoarginine behaves within the digestive tract like other amino acids: Homoarginine incorporated into dietary proteins is released during digestion and then absorbed at rates like those of other amino acids [49].
4. Homoarginine *per se *does not influence endogenous protein losses; the fact that HA behaves in the small intestine like a typical amino acid suggests that HA *per se* has no effect on endogenous protein output.
5. Homoarginine is not preferentially metabolised by gut microflora [31].
6. Homoarginine is easily and accurately determined by the ion-exchange chromatography as a typical amino acid during routine amino acid analysis without the need for specific hydrolysis or separation procedures.

Guanidination has been investigated in a diverse group of feed proteins, with widely differing lysine concentrations. The degree of conversion of lysine to HA varies greatly depending on the protein source, ranging from 83% to 100% for purified proteins, 40%–88% for common protein meals to 57%–63% for cereals [31, 50–54]. The factors affecting the extent of conversion are pH of the reaction mixture, the duration and temperature of incubation and, the ratio of O-methylisourea and lysine in the reaction mixture. The optimal guanidination conditions vary depending on the protein source and sometimes within the protein source.

It must be noted that, as per definition, only the endogenous loss of lysine is directly determined in the guanidination method [45]. The losses of other amino acids are then derived indirectly from based on the ratio of HA to amino acids in the guanidinated protein and ileal digesta. A special attribute of the HA method is that it can be used to determine the EAA secretions in animals given protein sources which contain fibre, anti-nutritional factors or both. This approach has been employed to measure total EAA losses in poultry [31, 55–57]. These studies confirm that ileal EAA losses determined by HA method are substantially higher than those determined by the protein-free diet. For example. Siriwan et al. [31] and Ravindran et al. [57] reported that values for EAA losses in broilers obtained using guanidinated casein were 2–3 times as much as those estimated either by feeding a nitrogen-free diet or by extrapolation to zero nitrogen intake.

Overall, in principle, the isotopic techniques and guanidination reaction are appealing for the measurement of total endogenous protein flows in animals that are in a physiologically normal state, following the feeding of wide range of protein sources. Both, however, suffer from practical limitations. Both are tedious, time-consuming and costly. Isotope dilution techniques require highly specialised personnel, equipment and facilities. Estimates from both approaches provide direct information only of the endogenous flow of one amino acid–lysine in the case of HA method and leucine in most cases of isotopes; flows of other amino acids are then calculated assuming a constant composition of endogenous protein but this assumption may not be valid for specific losses since, as discussed earlier, different anti-nutrients and fibre sources have diverging effects on individual components of the endogenous protein.

## Regression method

The regression method has been promoted in some studies for the direct measurement of true amino acid digestibility of feed ingredients [58–61]. It has also been suggested that the total (basal plus specific) ileal EAA losses can be determined under normal protein alimentation conditions using this method [58]. In this method, diets containing graded levels of AA from an ingredient are fed and a linear relationship between dietary nutrient input and their output in ileal digesta is established. This relationship permits the determination of diet independent theoretical total (basal plus specific) EAA losses and simultaneous measurement of true amino acid digestibility of the feed ingredient, as the intercept and slope of the regression, respectively.

Although, in theory, the true amino acid digestibility of the feed ingredient is directly determined, the laboriousness and cost involved limit its wider acceptance in nutritional research. Although the method overcomes the issue of physiological aberration, it incorrectly assumes that the EAA flow does not vary with the amount of protein fed [40]. Moreover, in several studies, regression method has yielded EAA estimates similar to that of protein-free diets [58] disputing its potential usefulness. A more important concern is the negative estimates reported for EAA loss in some studies. The notion that EAA losses can be negative is physiologically untenable and suggestive of the inherent limitation of the regression method [40]. Similar negative estimates for endogenous losses of phosphorus, with the regression method, have also been reported [62–64]. Overall, the negative estimates call the usefulness of this method to measure EAA losses into question.

## Data on specific ileal endogenous amino acid losses

The measurement of specific feed-induced EAA losses is both of practical as well as scientific interest, because of its consequences on intestinal dynamics, protein nutrition and energy costs [65]. However, published data on specific losses in poultry are non-existent, due largely to the complexity and specialised requirements of the methods to measure total EAA losses. Unpublished results from the Author’s group, summarised in Table 4, indicate that the specific EAA losses vary between feedstuffs, reflecting the effects of fibre and antinutrients on endogenous protein secretion.

**Table 4:** Specific feed-induced ileal endogenous protein losses (g/kg dry matter intake) in broiler chickens^a^

Feedstuff	Total losses^b^	Basal losses^c^	Specific losses^d^
Soybean meal, 480 g/kg crude protein	14	12	2
Soybean meal, 440 g/kg crude protein	21	12	9
Canola meal	28	12	16
Cottonseed meal	39	12	27

^a^University of Sydney, Camden, NSW, Australia, unpublished data

^b^Following feeding of guanidinated proteins

^c^Following feeding of enzyme-hydrolysed casein

^d^Total losses minus basal losses

## Influence of dietary components

While studies on total EAA losses induced by specific components present in native ingredients are limited, research to quantify the effects of added purified dietary components has attracted some attention [12]. Large numbers of studies have examined the influence of pure forms of fibre, lipids and individual anti-nutrients on EAA losses, by the addition of these compounds on top of diets designed to measure basal losses (e.g. protein-free diet, casein diet) or total losses (e.g. guanidinated casein, isotopic dilution). Select examples are summarised in Table 5 and the results of these studies, in general, confirm the EAA loss responses associated with a range of dietary factors.

**Table 5:** Studies evaluating the effects of pure forms of dietary components on ileal endogenous amino acid losses in broiler chickens – select examples

References	Background	Factors examined
Danicke et al. [36]	^15^N-isotope dilution	Lipids
Angkanaporn et al. [55]	Guanidinated casein	Non-starch polysaccharide
Siriwan et al. [66]	Guanidinated casein	Fibre level
Cowieson and Ravindran [67]	Enzyme-hydrolysed casein	Phytic acid
Cowieson et al. [68]	Enzyme-hydrolysed casein	Phytic acid
Kluth and Rodehutscord [69]	Regression method	Cellulose
Morel et al. [70]	Enzyme-hydrolysed casein	β-Glucan
Onyango et al. [71]	Glucose	Phytic acid
Adedokun et al. [72]	Casein -regression method	Dietary electrolytes
Adedokun and Applegate [73]	Protein-free diet	Dietary electrolytes
Adedokun et al. [74]	Protein-free diet	Calcium level
Whitehouse et al. [75]	Protein-free diet	Lipids

## Practical application of endogenous flow data

The quantification of ileal EAA flow is critical for better understanding of the efficiency of nitrogen utilisation. The endogenous nitrogenous components leaving the terminal ileum are lost to the animal and, represent a net cost to the environment and, importantly, to productivity and profitability. Measurement and comprehension of the factors influencing these losses provide an important practical strategy to enhance the efficiency of nitrogen use in the farm. These losses not only have direct caloric consequence for the digestible energy lost via endogenous amino acids [67, 68], but also ramifications on the energy cost associated with protein synthesis and cell turnover in the gut.

The prime application of the quantified EAA losses is for the correction of apparent digestibility to true digestibility values. It has now accepted that the ileal digestibility determined with growing birds are preferable over those determined at the total tract level. Accordingly, the apparent ileal digestibility must be corrected for ileal EAA losses and that the use of apparent values in feed formulations must be disbanded [76]. For reasons presented above, basal losses, as opposed to specific losses, are more relevant for such corrections. But the challenge is to agree on which basal EAA loss estimate should be considered. The question is whether to use the estimate from protein-free DMI or that from protein-containing DMI because diets always contain protein and the methods used for the determination of basal losses should use diets that contain protein [76, 77]. In the pig industry, there is consensus that a constant basal estimate based on the feeding of protein-free diets as being the valid value to correct for basal endogenous EAA losses [78] and the digestibility term ‘standardised’ has been put forward to replace the original term ‘true’ [79]. This approach has its drawbacks [77], but represents a way forward to overcome the limitations of apparent digestibility. In the poultry industry, on the other hand, there is less agreement on what should constitute basal EAA losses.

The resistance in the poultry industry to agree on a constant basal EAA value arises from the extreme variability found in published estimates following the feeding of protein diets (Table 6). Even after the exclusion of EAA outliers, the coefficient of variation was in excess of 30% for most amino acids. Many interrelated factors contribute to the reported variability. The main animal factors comprise of class of birds, poultry species, protein status, age, body weight and health status. The dietary factors include type of purified protein, indigestible dietary marker and energy source. Interestingly, there had been some debate and confusion about the influence of energy sources (starch and dextrose) on EAA. Marked influence of the ratio of starch and dextrose in assay diets was reported in one study EAA [88]. Curiously this study also reported extremely high EAA estimates (see Table 6). However, no such effects due to energy sources were seen in other studies [73] as well as in 16 studies in our laboratory. Methodological difference in the collection and processing of ileal digesta is one of the pivotal, and perhaps the most neglected, contributing factor as shown by Ravindran et al. [80]. Differences in analytical procedures and accuracy among laboratories (amino acids, marker) are equally, if not more, important but little-discussed causative factors.

**Table 6:** Reported variability in the basal endogenous flow (g/kg dry matter intake) in broiler chickens fed nitrogen-free diets^a^

Amino acids	Range of published values^a^	Average^b^	Coefficient of variation, %^b^
Aspartic acid	0.34–1.64	0.58	25.5
Threonine	0.27–0.97	0.50	18.4
Serine	0.26–1.03	0.45	24.1
Glutamic acid	0.42–2.13	0.77	27.6
Proline	0.24–1.07	0.43	45.4
Glycine	0.21–0.83	0.36	31.8
Alanine	0.14–0.91	0.30	28.5
Valine	0.21–1.01	0.37	25.4
Isoleucine	0.16–0.86	0.30	25.0
Leucine	0.25–1.45	0.43	30.2
Tyrosine	0.12–0.64	0.23	57.1
Phenylalanine	0.15–0.83	0.31	36.3
Histidine	0.07–0.42	0.15	37.2
Lysine	0.16–1.24	0.29	38.0
Arginine	0.16–1.02	0.29	32.8
Methionine	0.05–0.36	0.09	29.8
Cysteine	0.03–0.55	0.18	33.1

^a^References: Siriwan et al. [31]; Adedokun et al. [32]; Ravindran et al. [57]; Adedokun et al. [72]; Adedokun et al. [73]; Adedokun et al. [74]; Ravindran et al. [80]; Adedokun et al. [81]; Adedokun et al. [82]; Adedokun et al. [83]; Golian et al. [84]; Ravindran et al. [85]; Woyengo et al. [86]; Soleimani et al. [87]; Kong and Adeola [88]; Ravindran et al. [89]; Toghyani et al. [90]; Cowieson et al. [91]; Osho et al. [92]; An et al. [93]; Cowieson et al. [94]

^b^Average values and variability were calculated after removal of the following extreme outliers: Adedokun et al. [32]; Adedokun et al. [73]; Kong and Adeola [88]

In the author’s opinion, despite its inherent limitations, the protein-free diet presents the best strategy among available techniques for the measurement of basal EAA losses. There may be four ways to address the impasse in the poultry industry on the agreement of a constant basal estimate. First, to measure basal losses in each digestibility assay. This will add to the cost and ideally must be avoided, but will suit research stations where digestibility assays are not carried out routinely. Second, since the assay conditions (assay diet composition, bird age, strain, housing etc.) are standardised within a research station and the variability will be minimal, average the basal EAA estimates across assays or over years and use this station-specific value for the correction. This approach will suit stations where the conduct of amino acid digestibility assays is routine. Third, collate available published data, remove outliers using appropriate statistical models and agree on average values that can be used globally as has been done in the pig industry. Finally, to conduct a collaborative ‘ring test’ involving several key research stations using pre-agreed standardised assay conditions and controls, as has been conducted for ileal amino acid digestibility assay [80], and develop estimates that could be used globally.

## Terminology used to describe digestible amino acids

Before concluding, a note on the use of AA digestibility terminology in relation to different estimates of endogenous losses is relevant. In recent decades, the digestible amino acid system used by the poultry industry has shifted from excreta values to ileal-based values and several compilations of ileal amino acid digestibility of feed ingredients are now available [95–97]. Considerable confusion, however, still exists regarding the ileal digestibility terminologies used in the literature – apparent, true, standardised and real. In particular, the terms true vs. standardised digestibility are being used interchangeably in recent times and different definitions are being presented by different groups, creating confusion among end-users. For practical purposes, true and standardised digestibility refer to the same. In the pig industry, the use of the term ‘standardised ileal digestibility (SID)’ has become the norm [79] and also being increasingly used in poultry.

A further confusion arises from the use, by some researchers, of the term real digestibility, which includes the correction for the total endogenous losses and is related to the feeding of a specific ingredient [10, 41]. Real digestibility may be more reliable than true digestibility [98], but (as noted above) at the current time insufficient data are available on total EAA losses to seriously consider this system. It is suggested that this issue is probably more theoretical rather than practical and the merit of real versus true digestibilities in applied nutrition is questionable. For a detailed discussion on digestible amino acid systems, readers are referred to Moughan et al. [77].

## Conclusions

Endogenous protein losses represent significant protein and energy maintenance costs to the animal. Quantification of these losses is clearly relevant to improve the efficiency of protein digestion and for the better understanding of the factors influencing these losses. The progress in the measurement and factors influencing the ileal endogenous amino acid flow in poultry was examined in this review. During the 1980’s and 1990’s, there was heightened research activity on the evaluation of isotopic and homoarginine techniques to better comprehend the insights of endogenous protein flow in animals. For unknown reasons, this interest has in EAA, in general, slowed down in recent years. All the techniques reported in this review have their own merits and limitations, and none are perfect. The present review is the first report identifying taurine as a component amino acid in the endogenous protein flow in poultry. It is recommended that future poultry assays on EAA losses include estimates for taurine. An array of techniques has been tested and evaluated to replace the protein-free diet for EAA flow measurements. Continued use of this century-old method [2] at the present time may seem archaic. Until other research techniques are refined and simplified, however, protein-free diets will continue to provide information to resolve important questions about EAA and protein nutrition. As seen, endogenous protein flow is a complex issue and one that deserves critical thinking and investigation.

## Data Availability

Not applicable.

## Abbreviations

DMI: Dry matter intake
EAA: Endogenous amino acids
HA: Homoarginine
SID: Standardised ileal digestibility
